# Flexor Hallucis Longus tendon rupture in RA-patients is associated with MTP 1 damage and pes planus

**DOI:** 10.1186/1471-2474-8-110

**Published:** 2007-11-06

**Authors:** Henriette Baan, Wiepke K Drossaers-Bakkers, Rosemary Dubbeldam, Jaap J Buurke, Anand Nene, Martin AFJ van de Laar

**Affiliations:** 1 Ziekenhuis Groep Twente, Almelo & Hengelo, The Netherlands; 2 Medisch Spectrum Twente & University Twente, Enschede, The Netherlands; 3 Roessingh Research and Development, Enschede, The Netherlands

## Abstract

**Background:**

To assess the prevalence of and relation between rupture or tenosynovitis of the Flexor Hallucis Longus (FHL) tendon and range of motion, deformities and joint damage of the forefoot in RA patients with foot complaints.

**Methods:**

Thirty RA patients with painful feet were analysed, their feet were examined clinically for the presence of pes planus and range of motion (ROM), radiographs were scored looking for the presence of forefoot damage, and ultrasound examination was performed, examining the presence of tenosyovitis or rupture of the FHL at the level of the medial malleolus. The correlation between the presence or absence of the FHL and ROM, forefoot damage and pes planus was calculated.

**Results:**

In 11/60(18%) of the feet, a rupture of the FHL was found. This was associated with a limited motion of the MTP1-joint, measured on the JAM (χ^2 ^= 10.4, p = 0.034), a higher prevalence of pes planus (χ^2 ^= 5.77, p = 0.016) and a higher prevalence of erosions proximal at the MTP-1 joint (χ^2 ^= 12.3, p = 0.016), and joint space narrowing of the MTP1 joint (χ^2 ^= 12.7, p = 0.013).

**Conclusion:**

Rupture of the flexor hallucis longus tendon in RA-patients is associated with limited range of hallux motion, more erosions and joint space narrowing of the MTP-1-joint, as well as with pes planus.

## Background

In rheumatoid arthritis (RA), synovial inflammation affects the joints as well as periarticular structures such as tendons. It is well recognized that the inflammatory tissue in rheumatoid arthritis has a local destructive potency. Inflammation and the resulting damage both cause functional limitations [1]. Tenosynovitis or rupture of the tendon of the tibialis posterior is considered prevalent and important in the development of foot deformities in the feet of RA patients [2,3]. Rupture or tenosynovitis of the flexor hallucis longus (FHL) is rarely recognized by clinical examination in RA. This may be due to underestimation, since in clinical assessment of the painful hind foot, swelling is often interpreted as synovitis of the ankle [4]. Imaging has shown to be more sensitive in detecting tenosynovitis than physical examination [2,4,5]. MRI-studies in RA-patients with hind foot pain showed a FHL-tenosynovitis prevalence of approximately 20% [2,4]. Ultrasound studies (US) in RA patients showed a higher prevalence of FHL tenosynovitis then anticipated clinically [5,6]. To the best of our knowledge, FHL rupture is never reported in RA-patients.

Considering its function, damage of the flexor hallucis longus (FHL) as a possible consequence of tenosynovitis might be relevant. The FHL not only flexes the great toe but it contributes, together with plantar fascia, to the distribution of forces at the plantar side of the forefoot and maintenance of the longitudinal arch of the foot [7]. Loss of the tendon and its loading capability of the longitudinal arch, esp. at the level of the first ray, can lead to a pes planus [8].

Tenosynovitis (or rupture) of this tendon can also result in a (functional) hallux rigidis and tightening of the FHL tendon. The subsequent dorsal compression in the first MTP-joint can in turn lead to the forming of osteophytes, further mechanical impingement, limitation and damage of the MTP1 [9].

The relation between FHL tenosynovitis or rupture and aforementioned abnormalities of the foot in RA patients has to be determined.

In our study we aim to assess the prevalence of FHL tenosynovitis or rupture and the relation between FHL rupture or tenosynovitis and the range of motion, joint damage and pes planus in symptomatic feet of RA-patients.

## Methods

We included 30 consecutive RA patients with at least one painful forefoot and or hind foot who visited the outpatient rheumatology clinic of the Medisch Spectrum Enschede in September 2005. In 60 feet we measured the range of motion of the first metatarsophalangeal joint using the joint alignment motion scale (JAM) [10]. A normal range of motion (ROM) of MTP is scored 0. A ROM limitation to 65–70 degrees is scored 1, to 55–65 degrees as 2, to 20–55 degrees as 3 and a range of motion less then 20 degrees is scored as 4. Spiegel et al described the JAM scale and this shows good inter reader reliability as well as a good relation with disease activity and function [10,11].

The feet were examined clinically for the presence or absence of a pes planus.

Radiographs were made of all feet. The MTP and IP joints were each scored for joint erosions (range 0–10) and joint space narrowing (0–4) per joint according to Sharp/van der Heijde [12].

Erosion score of the proximal surface of MTP1 (0–5) was scored separately, as FHL tendon problems can lead to a hallux rigidis with a higher prevalence of dorsal erosions of the first metatarsal head [9].

One licensed and qualified rheumatologist, using a Logiq 7 General Electrics, 7–13 MHz linear transducer, performed ultrasound investigation. If present, the FHL tendon cross section was measured and the tendon assessed for signs of tenosynovitis (fluid around the tendon or presence of Power Doppler signs). This was performed at the level of the medial malleolus and extended 6 cm proximal to 6 cm distal of this point. Rupture of the FHL tendon was defined as absence of this tendon at the level of the medial malleolus. This tendon is difficult to visualise. If it could not be found at first sight, the great toe was flexed, causing motion of the tendon. If no motion was detected, the FHL tendon was finally judged to be absent.

Ultrasound is regarded as a reliable tool for detecting tendon abnormalities. Naredo et al observed an overall agreement of 88.5% in detecting tenosynovitis and 92% in tendon lesions of the ankle and foot, although these findings were not limited to the tendon of the FHL. Scheel et al observed an excellent κ value of 1 for the detection of tendon tears and a moderate κ value of 0.49 in detecting tenosynovitis, but this was calculated for tendons in general and not specified for the tendons of the ankle or the FHL [13,14].

Differences between groups, regarding the rupture of the FHL, and the correlation with the ROM, Sharp/van der Heijde score and presence of pes planus were tested, using Pearson's chi squared test. Ethical approval was obtained from the ethics committee from The Medical Spectrum Twente; all patients gave their written consent.

## Results

Table 1 shows the demographic characteristics of the studied RA patients, showing a wide range of age, disease duration and damage.

**Table 1:** Mean values of the demographic, radiographic and joint mobility characteristics.

	**TOTAL N = 60**	**FHL TENDON ABSENT N = 11**	**FHL TENDON PRESENT N = 49**	**PEARSON'S χ (P VALUE)**
**Age **(years)	54	57.6	53.1	NS
**Disease duration **(years)	11.6	11.2	11.7	NS
**JAM score MTP-1 motion (0–4)**	2.1	3.09	1.84	10.4 (0.034)
**SHS erosion proximal MTP 1 (0–5)**	1.0	2.18	0.74	12.3 (0.016)
**SHS narrowing MTP 1 (0–4)**	1.62	2.55	1.4	12.7 (0.013)
**Total SHS feet (0–84)**	29.7	47.1	25.6	NS
**Pes Planus**	42	11/11	31/49	5.77 (0.016)

JAM = joint alignment motion scale, SHS = Sharp van der Heijde score, FHL = flexor hallucis longus

The median and the range of the JAM score are provided in Table 1.

Forty-two feet (70%) were scored as a pes planus.

Results of the radiograph scores are presented in Table 1.

Ultrasonography revealed that the tendon of the FHL was ruptured in 11/60(18%) of the feet Figure 2.

FHL tendon rupture was associated with a limited range of motion of MTP1, measured as a significant higher score of the JAM motion MTP1 (χ^2 ^= 10.4, p = 0.034.)

In only one foot, a tenosynovitis was diagnosed, based on fluid around the tendon. No tendon tears were found.

A pes planus was found in all of the feet with a ruptured FHL, and only in 31 of the 49 remaining feet (χ^2 ^= 5.77, p = 0.016.)

There was a significant relation between rupture of the FHL and erosions proximal at the MTP-1 joint (χ^2 ^= 12.3, p = 0.016.), and joint space narrowing of the MTP1 joint. (χ^2 ^= 12.7, p = 0.013.)

## Discussion

This study shows that in RA patients, rupture of the flexor hallucis longus tendon is associated with limited range of hallux motion, erosions and joint space narrowing of the MTP-1 joint and pes planus.

The observed prevalence of FHL rupture seems high in this study. We must stress that this is not representative for the RA population since we included only patients with a painful foot. However, the reported prevalence of FHL tenosynovitis in the study of Maillefert et al is also rather high, 3/17 feet (18%) [4]. As far as we know, there are no reports on the prevalence of ruptured FHL in RA patients.

**Figure 1:**
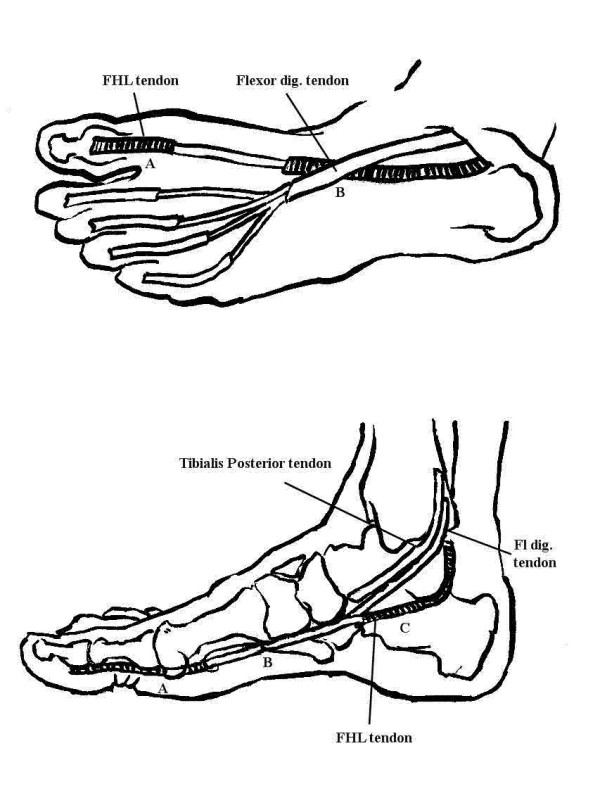
a. Medioplantar aspect of the foot. b. Medial aspect of the foot

FHL rupture (following tenosynovitis) might be provoked by rheumatoid inflammation. As stated earlier, RA can affect tendons as well as joints. This can occur at the level A and C in the Figures 1a and 1b. Early damage of the great toe joint might lead to limited joint motion and subsequent chronic underuse of the FHL, contributing to atrophy of its tendon [15].

Mechanical reasons for inflammation or rupture of the tendon are deformity or anatomical variations of the foot at the level B or C, Figure 1, as can be seen in calcaneus fractures or bony abnormalities like a prominent os trigonum, or overuse of the tendon in runners, dancers and athletes [16].

Although nothing can be concluded regarding causality, we hypothesize that following rheumatoid inflammation, rupture of the FHL tendon takes place. This can occur unnoticed, as the associated pain and swelling of the ankle are often erroneously contributed to synovitis of the ankle [4]. During tenosynovitis, damage of the MTP-1 may arise, according to the mechanism described by Michelson et al [9].

The association with a pes planus can be explained by the loss of the FHL in its supporting role of distributing the forces (together with the fascia plantaris) under the foot and maintenance of the longitudinal arch, as described by Hamel et al [7].

We hypothesize that early recognition and timely adequate treatment of tenosynovitis of the FHL (for example by local ultrasound guided steroid injections) might become important to prevent damage.

A larger prospective follow-up study however, demonstrating the causal relationship between tenosynovitis or rupture of the FHL and deformities in the rheumatoid foot is warranted to draw definite conclusions.

## Conclusion

Rupture of the flexor hallucis longus tendon in RA-patients is associated with limited range of hallux motion, more erosions and joint space narrowing of the MTP-1-joint, as well as with pes planus.

## Competing interests

The author(s) declare that they have no competing interests.

## Authors' contributions

HB conceived of the study, carried out the study and drafted the manuscript.

WD and ML conceived of the study, analysed and interpreted the data and drafted the manuscript.

AN, JB and RD analysed and interpreted the data.

All authors read and approved the final manuscript.

**Figure 2:**
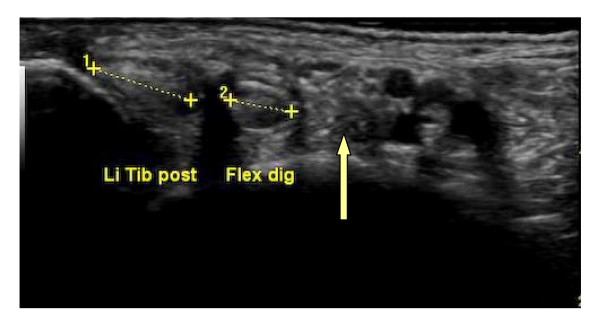
Left medial ankle of a 57-year-old patient with RA, missing the FHL tendon(arrowhead).

## Pre-publication history

The pre-publication history for this paper can be accessed here:


